# Variants in *JAZF1* are associated with asthma, type 2 diabetes, and height in the United Kingdom biobank population

**DOI:** 10.3389/fgene.2023.1129389

**Published:** 2023-06-12

**Authors:** Andrew T. DeWan, Megan E. Cahill, Diana M. Cornejo-Sanchez, Yining Li, Zihan Dong, Tabassum Fabiha, Hao Sun, Gao Wang, Suzanne M. Leal

**Affiliations:** ^1^ Department of Chronic Disease Epidemiology and Center for Perinatal, Pediatric and Environmental Epidemiology, Yale School of Public Health, New Haven, CT, United States; ^2^ Center for Statistical Genetics, Gertrude H. Sergievsky Centerand the Department of Neurology, Columbia University Medical Center, New York, NY, United States; ^3^ Department of Biostatistics, Yale School of Public Health, New Haven, CT, United States; ^4^ Taub Institute for Alzheimer’s Disease and the Aging Brain, Columbia University Medical Center, New York, NY, United States

**Keywords:** asthma, type 2 diabetes, height, pleiotropy, genome-wide association study, mediation analysis, fine-mapping, United Kingdom Biobank

## Abstract

**Background:** Asthma, type 2 diabetes (T2D), and anthropometric measures are correlated complex traits that all have a major genetic component.

**Objective:** To investigate the overlap in genetic variants associated with these complex traits.

**Methods:** Using United Kingdom Biobank data, we performed univariate association analysis, fine-mapping, and mediation analysis to identify and dissect shared genomic regions associated with asthma, T2D, height, weight, body mass index (BMI), and waist circumference (WC).

**Results:** We found several genome-wide significant variants in and around the *JAZF1* gene that are associated with asthma, T2D, or height with two of these variants shared by the three phenotypes. We also observed an association in this region with WC when adjusted for BMI. However, there was no association with WC when it was not adjusted for BMI or weight. Additionally, only suggestive associations between variants in this region and BMI were observed. Fine-mapping analyses suggested that within *JAZF1* there are non-overlapping regions harboring causal susceptibility variants for asthma, T2D, and height. Mediation analyses supported the conclusion that these are independent associations.

**Conclusion:** Our findings indicate that variants in the *JAZF1* are associated with asthma, T2D, and height, but the associated causal variant(s) are different for each of the three phenotypes.

## Introduction

Asthma, Type 2 Diabetes (T2D), and anthropometric measurements are all complex traits and are often correlated, suggesting there are shared etiological factors. The global prevalence of individuals with a current asthma diagnosis is estimated to be 262 million, with 37 million incident cases each year. (Vos et al., 2020). As of 2012, prevalence of asthma in the United Kingdom (United Kingdom) is ∼8%, with ∼12% of the population ever having an asthma diagnosis. (Strachan et al., 2017). Globally, there are an estimated 438 million cases of T2D, (Vos et al., 2020), and more than four million cases in the United Kingdom. (Whicher et al., 2020) Cases of obesity, defined as a body mass index (BMI) greater than 30, have been increasing globally, with 26% of men and 29% of women in England classified as obese. (Team, 2020). Studies have shown individuals with both obesity and asthma are more likely to have severe asthma that is poorly controlled, (Lavoie et al., 2006; Taylor et al., 2008; Juel and Ulrik, 2013), and one meta-analysis found individuals with both asthma and T2D also had poorer control of asthma. (Torres et al., 2021).

Obesity is a well-established risk factor for both asthma and T2D. (Barnes, 2011; Ali and Ulrik, 2013; Shore, 2013). While highly correlated, waist circumference (WC) can provide distinct information on adiposity as it is a measure of visceral obesity, specifically WC adjusted for BMI. WC is often used in studies of chronic diseases. (Jacobs et al., 2010; Darsini et al., 2020). Increased WC has been shown to be an additional risk factor for T2D (Feller et al., 2010) and asthma (Von Behren et al., 2009) even after adjusting for BMI. Elevated blood glucose and T2D have been linked to increased risk of asthma in adults, (Brumpton et al., 2013), and conversely, asthma has been associated with increased risk of developing T2D in adults. (Mueller et al., 2013). These epidemiological studies suggest a biological link between asthma, T2D, and WC, although the underlying mechanisms of these comorbidities are not completely understood, (Perez and Piedimonte, 2014), there is evidence pointing to shared genetic variants. (Zhu et al., 2020).

Height is a highly heritable polygenic trait. (Yang et al., 2010). There is evidence that shorter individuals have an increased risk for developing T2D (Shrestha et al., 2019) and individuals with childhood onset asthma have shorter stature as adults compared to non-asthmatics. (Chen et al., 2022).

Genetic variants have been well documented to influence each of these phenotypes, (Xue et al., 2018; Kim and Ober, 2019; Pulit et al., 2019; Yengo et al., 2022), and genetic pleiotropy, wherein variants affect more than one trait or disease, could be an additional cause of disease comorbidity. (Visscher et al., 2017). By identifying cross-phenotype associations, there is the potential to identify novel genetic links between phenotypes and new insight into the underlying mechanisms of known comorbid conditions.

To identify whether variants contribute to asthma, T2D, and anthropometric measurements, we analyzed the United Kingdom Biobank dataset (Sudlow et al., 2015), studying genetic and phenotypic data collected on White Europeans. We were able to identify cross-phenotype associations for three of our phenotypes of interest (asthma, T2D, and height), identifying variants in *JAZF1* that were significantly associated with all three phenotypes. Fine-mapping analysis, suggested that the underlying causal variants are not shared between asthma, T2D, and height. This finding was also supported by mediation analysis.

## Methods

*Data access and ethical approval.* This research was conducted using the United Kingdom Biobank Resource (application number 32285). The United Kingdom Biobank study was conducted under generic approval from the National Health Services’ National Research Ethics Service. The present analyses were approved by the Human Investigations Committee at Yale University (2000026836) and by the Institutional Review Board at Columbia University (IRB-AAAS3494).

*Discovery Dataset*. Participants were enrolled in the United Kingdom Biobank, and data accessed under an approved agreement (application ID 32285). Genotypes were assayed on either the United Kingdom BiLEVE array or the United Kingdom Biobank Axiom array with 733,332 autosomal variants overlapping between the two arrays. Subject and variant quality control is described in Supplementary Table S1, with subjects limited to those who self-defined as White British (field 22006) that have very similar genetic ancestry based on a principal components (PCs) analysis of their genotypes and have >99% of their genotypes (N = 366,752) (Supplementary Figure S1). For the analysis only variants with a call rate >99%, that did not deviate from Hardy-Weinberg Equilibrium (*p*-value > 5 × 10^−8^), and minor allele frequency (MAF) > 0.01 were included (N = 529,024). Additionally, imputed variants provide by the United Kingdom Biobank that had a MAF >0.001 and Info Score >0.8 were analyzed (N = 13,407,279). All variants were annotated using ANNOVAR. (Wang et al., 2010).

For analyses requiring unrelated individuals, we limited the dataset of White British by using the indicator variable (field 22020) to obtain study subjects that are unrelated (more distantly related than third degree relatives). This resulted in a subset of 307,259 unrelated individuals for analysis (Supplementary Figure S1).

*Replication Dataset*. For the replication sample, we identified a set of non-British White Europeans using self-reported ancestry and pre-computed PCs (field 22009). First, the mean and covariance were calculated for 40 PCs from all genetically confirmed White British subjects. For each subject, the Mahalanobis distance from the empirical PC distribution was calculated. Among all 488,377 United Kingdom Biobank subjects prior to any exclusions, 409,615 are self-reported White British (field 22006); 50,518 are self-reported White Europeans but who are not British (field 21000). After quality control, the final replication dataset consisted of 44,173 individuals who are non-British White Europeans (Supplementary Figure S1) with 13,243,888 imputed and 550,028 directly genotyped variants.

*Phenotype Definitions*. The T2D phenotype was defined by either ICD-10 code (United Kingdom Biobank field 41270, code E11) or self-reported diagnosis by a doctor at ≥ 30 years of age (fields 2,443 and 2,976). Individuals with type 1 diabetes [self-reported diabetes that occurred <30 years of age or E10] or gestational diabetes [self-report (field 4,011) or O24] were excluded from both cases and controls. Asthma was defined by either ICD-10 code (field 41270, J45 or J46) or self-reported diagnosis by a doctor (field 6,152). Individuals with autoimmune conditions were excluded from both the asthma and T2D controls [field 20002, self-reported diagnosis of an autoimmune disease, (Harpsoe et al., 2014), or self-report of sarcoidosis diagnosis by doctor in field 22133]. Individuals who did not report a diagnosis of asthma or T2D at the recruitment visit but did report a diagnosis at a later visit were also excluded from both the cases and controls for that phenotype. In the discovery dataset, for T2D, there were 22,670 cases and 313,404 controls, and for asthma, there were 48,623 cases and 290,722 controls. In the replication dataset, for T2D, there were 2,625 cases and 39,324 controls, and for asthma, there were 5,822 cases and 36,615 controls. WC (field 48) excluded women pregnant at time of recruitment (field 3,140), for a total of 365,499 subjects included in the discovery WC analysis and 43,954 subjects in the replication analysis. Height was obtained from field 50 (N = 365,934 discovery and N = 44,010 replication) and weight from field 21002 (N = 365,703 discovery and N = 43,976 replication). BMI was obtained from field 21001 (N = 365,498 discovery and N = 43,966 replication) which was computed from height (field 50) and weight (field 21002). When used as outcomes, WC, BMI, height, and weight were transformed using rank-based inverse normal transformation as implemented in R. A summary of the phenotypes, age, and sex for the individuals included in the discovery and replication datasets is provided in Table 1.

**TABLE 1:** Discovery and replication sample demographics.

		Asthma cases	Asthma controls	T2D cases	T2D controls	WC (cm)	BMI (kg/m^2^)	Height (cm)	Weight (kg)
Discovery	N/mean (sd)	48,623	290,722	22,670	313,404	90.33 (14.46)	27.42 (4.76)	168.71 (9.23)	78.26 (15.87)
	Sex (N [%] male)	20,483 (42.13)	135,525 (46.62)	14,202 (62.65)	141,147 (45.04)	167,655 (45.87)	167,654 (45.87)	167,854 (45.87)	167,748 (45.87)
	Age at recruitment (mean [sd])	56.48 (8.18)	56.89 (7.98)	60.47 (6.68)	56.60 (8.04)	56.91 (8.00)	56.92 (8.00)	56.92 (8.00)	56.92 (8.00)
Replication	N/mean (sd)	5,822	36,615	2,625	39,324	89.74 (13.75)	27.28 (4.92)	168.36 (9.21)	77.56 (16.25)
	Sex (N [%] male)	2,454 (42.15)	16,252 (44.39)	1,610 (61.33)	16,936 (43.07)	19,379 (44.09)	19,380 (44.08)	19,391 (44.06)	19,389 (44.09)
	Age at recruitment (mean [sd])	55.26 (8.30)	55.40 (8.16)	59.34 (7.06)	55.13 (8.17)	55.44 (8.17)	55.44 (8.17)	55.44 (8.17)	55.44 (8.18)

*Phenotype Specific Principal Components Analyses*. Using the directly genotyped variants, the data was pruned for linkage disequilibrium (LD) using the default parameters of PLINK1.9 (Purcell et al., 2007) (50 variant window, pairwise *r* ^2^ < 0.2, and shifting by 5 variants after each step). PCs analysis was performed for each trait separately using the smartPCA program in EIGENSOFT-7.2.1. (Patterson et al., 2006). These PCs were included in the univariate analysis.

*Univariate Analyses*. Univariate association analysis was performed on the discovery and replication samples using the full set of imputed and directly genotyped variants using linear mixed models (LMM) and generalized (LMM) as implemented in REGENIE. (Mbatchou et al., 2021). REGENIE implements a multi-stage procedure which starts by fitting a null model with whole genome regression, which is an approximation of the LMM/GLMM, using ridge regression on the directly genotyped variants to estimate the polygenic effects parameter to account for relatedness and population stratification and subsequently using the full set of imputed variants to estimate the genetic prediction of each phenotype using a leave one chromosome out procedure. For binary traits, Firth regression is used to ensure that the results are well calibrated in the presence of low frequency variants and unbalanced case-control ratios, both of which are present in this analysis. REGENIE implements an approximate Firth regression so that this approach can be run on a genome-wide scale. For the analysis of all phenotypes [asthma, T2D, WC adjusted for BMI (WCadjBMI), WC, height, weight, and BMI], age at recruitment (field 21022), genetic sex (field 22001), and 10 PCs were included in the regression model. Due to the well-known associations in the HLA regions with these phenotypes, variants in the HLA region were not considered for further evaluation. LD clumping of the univariate association results from the discovery sample was performed for asthma, T2D, and height in PLINK using a 4 MB region surrounding the index variant and a *r* ^2^ threshold of 0.2 to identify overlapping genome-wide significant (*p* ≤ 5 × 10^−8^) signals across the three phenotypes. LD clumping takes into account the correlation structure between variants due to LD and significance level to obtain a set of independently associated loci.

*Region visualization.* Using LocusZoom (Boughton et al., 2021) regional plots were created from summary statistic data obtained for each phenotype for the discovery sample. The most significant variant from each phenotype association analysis was used as the reference variant in each of the three plots. For the eight cross-phenotype associated variants, *r* ^2^ values were calculated using the genetic data obtained from the unrelated subjects and plotted using Haploview. (Barrett et al., 2005).

*Fine-mapping.* To identify a set of causal independent variants for asthma, T2D, and height, we used the Sum of Single Effects (SuSiE) model. (Wang et al., 2020). SuSiE identifies credible sets in a region that contain independent or highly correlated variants with nonzero effects by implementing an iterative Bayesian stepwise selection procedure. Fine-mapping was performed on the *JAZF1* region using variants that were selected via LD clumping (see Univariate Analyses). The summary statistics and genotypes for these regions were extracted and the sample and population LD matrices were calculated using in-house workflows. For fine-mapping, we assumed a maximum of 10 casual variants with non-zero effects and identified the 95% credible sets using the summary statistics and LD matrices obtained for the discovery sample.

*Mediation analysis.* To address whether or not the cross-phenotype associations could be explained by mediation through one or more of the phenotypes, mediation analysis was conducted using the *Mediation* R package. (Tingley et al., 2014). The two variants in *JAZF1* showing cross-phenotype associations for all three phenotypes were included. Given the temporal ordering of the phenotypes, with T2D in general having a later onset than asthma in the majority of our subjects (average age of diagnosis for T2D was 53.6 years and for asthma was 31.3 years, and in subjects with both T2D and asthma, the T2D diagnosis was an average of 20.5 years after the asthma diagnosis), we considered T2D to be the outcome and asthma the mediator when considering those two phenotypes in the mediation analysis. Despite WC being measured at baseline in UKB, for consistency with the T2D and asthma mediation analysis, we considered T2D as the outcome and height as the mediator. Since *Mediation* can only use standard linear or logistic regression, we only included the unrelated subjects (N = 307,259) and among those, subjects that had both asthma and T2D (N = 302,457) and height and T2D (N = 300,329) data. We performed mediation analyses using the two SNPs in *JAZF1* that were genome-wide significant for asthma, T2D, and height. Each mediation analysis aimed to decompose the total effect of the SNP on T2D into direct and indirect effect estimates. Direct and indirect SNP effects were estimated as previously described. (Imai et al., 2010). Ninety-five percent confidence intervals for all effect estimates are bias corrected and accelerated intervals (DiCiccio and Efron, 1996) estimated via nonparametric bootstrapping. A significance threshold of *α* = 0.05 was used to evaluate the significance of the total, direct, and indirect effects.

## Results

**FIGURE 1:**
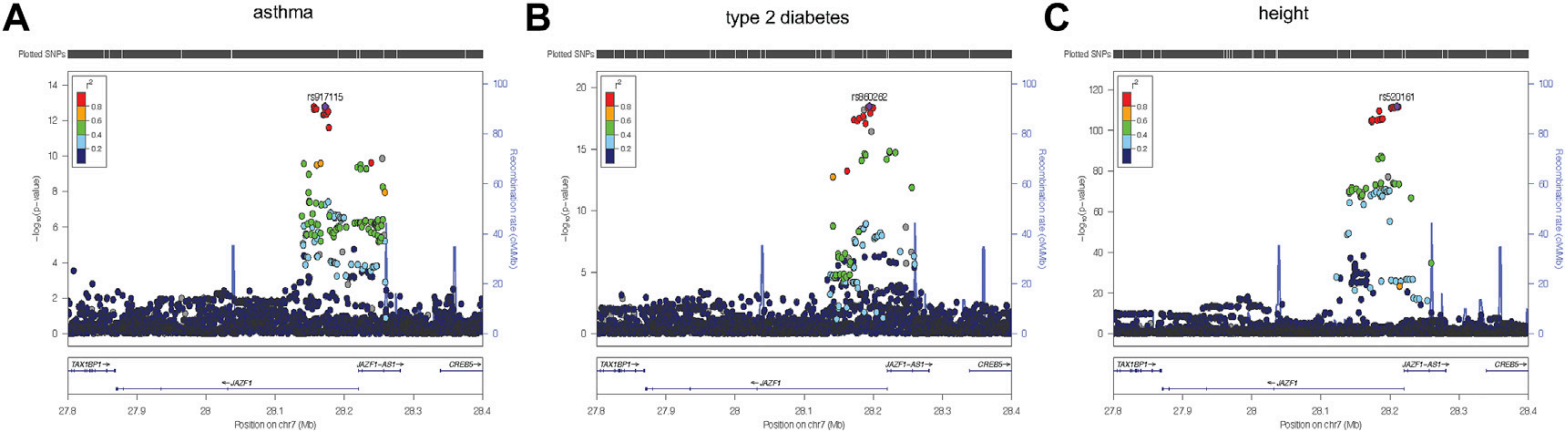
Regional plots were created using LocusZoom (Boughton et al., 2021) from the discovery association results for asthma **(A)**, type 2 diabetes **(B)**, and height **(C)**. The most significantly associated variant for each of the three phenotypes, was selected as the reference variant and is indicated with purple diamond in each plot. Linkage disequilibrium (as measured by r_2_) was estimated from the data. It is evident from each plot that the strongest association signal for each phenotype is within *JAZF1* rather than *JAZF1-AS1*.

**FIGURE 2:**
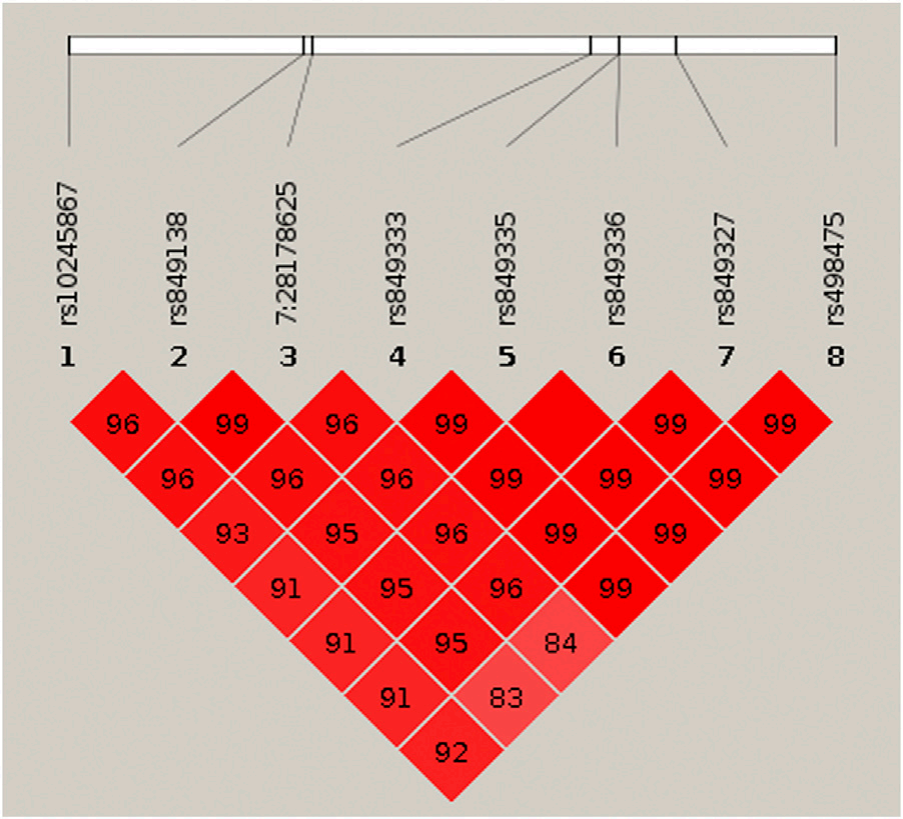
HaploView plot for the eight genome-wide significant cross-phenotype associated variants in and around *JAZF1*. Pairwise linkage disequilibrium (as measured by r^2^) is displayed for the eight genome-wide significant variants (see Table 2). Where an r^2^ value is not indicated, r^2^ = 1.0. The plot was created in HaploView (Barrett et al., 2005) using the subset of unrelated subjects.

To identify variants potentially displaying biological pleiotropy across asthma, T2D, and several anthropometric measures, we first conducted a genome-wide association study for each phenotype (Supplementary Figure S2). We then performed LD clumping, as described in the Methods, to identify independent regions and variants associated with asthma T2D and anthropometric measurement (height, weight, BMI, WCadjBMI, WC). We filtered variants from the association results that had genome-wide significant *p*-values (≤5 × 10^−8^) in for asthma, T2D and at least one anthropometric measure. Outside of the HLA region, only one region, located on chromosome 7, contained eight variants that were genome-wide significant for at least three of the phenotypes (Table 2). The variants with the smallest *p*-values in this region were rs10245867 for asthma, rs849138 for height, 7:28178625 for T2D, and WCadjBMI. These variants are in the coding region of the *JAZF1* and *JAZF1* antisense RNA 1 (*JAZF1-AS1*) genes (Figure 1). Across this region, all eight variants were in relatively high LD, with *r* ^2^ values ranging from 0.84 to 1.00 (Figure 2).

**TABLE 2:** Variants in *JAZF1* with genome-wide significant associations with asthma, T2D and at least one anthropometric measure.

Variant	BP^a^	EA^b^	EAF^c^	INFO^d^	Asthma				T2D				WCadjBMI				WC			
					Discovery		Replication		Discovery		Replication		Discovery		Replication		Discovery		Replication	
					Beta	*p*-value	Beta	*p*-value	Beta	*p*-value	Beta	*p*-value	Beta	*p*-value	Beta	*p*-value	Beta	*p*-value	Beta	*p*-value
rs10245867	28142186	T	0.329	0.995	0.0471	2.8 × 10^−10^	0.0642	2.7 × 10^−03^	0.0635	1.9 × 10^−09^	0.0955	2.2 × 10^−03^	0.0058	3.4 × 10^−08^	0.0122	6.3 × 10^−05^	−0.0006	7.8 × 10^−01^	0.0089	1.5 × 10^−01^
rs849138	28177338	G	0.493	0.998	0.0387	4.0 × 10^−08^	0.0398	5.1 × 10^−02^	0.0863	5.2 × 10^−18^	0.0906	2.3 × 10^−03^	0.0119	1.3 × 10^−32^	0.0192	3.2 × 10^−11^	0.0036	6.8 × 10^−02^	0.0157	6.6 × 10^−03^
7:28178625	28178625	TG	0.493	0.997	0.0394	2.3 × 10^−08^	0.0410	4.4 × 10^−02^	0.0860	6.7 × 10^−18^	0.0922	1.9 × 10^−03^	0.0120	7.7 × 10^−34^	0.0188	8.2 × 10^−11^	0.0036	7.0 × 10^−02^	0.0151	9.3 × 10^−03^
rs849333	28219812	A	0.342	0.996	0.0461	4.5 × 10^−10^	0.0502	1.9 × 10^−02^	0.0813	7.2 × 10^−15^	0.0765	1.4 × 10^−02^	0.0067	1.7 × 10^−10^	0.0115	1.5 × 10^−04^	0.0000	9.8 × 10^−01^	0.0081	1.8 × 10^−01^
rs849335	28223990	T	0.341	N/A	0.0464	3.3 × 10^−10^	0.0519	1.5 × 10^−02^	0.0828	2.0 × 10^−15^	0.0755	1.5 × 10^−02^	0.0069	4.3 × 10^−11^	0.0105	4.9 × 10^−04^	0.0004	8.4 × 10^−01^	0.0064	2.9 × 10^−01^
rs849336	28224053	A	0.341	1.000	0.0458	5.5 × 10^−10^	0.0531	1.3 × 10^−02^	0.0831	1.6 × 10^−15^	0.0757	1.5 × 10^−02^	0.0069	4.2 × 10^−11^	0.0105	4.9 × 10^−04^	0.0005	8.2 × 10^−01^	0.0064	2.9 × 10^−01^
rs849327	28232457	A	0.341	0.997	0.0459	5.4 × 10^−10^	0.0527	1.4 × 10^−02^	0.0829	2.0 × 10^−15^	0.0782	1.2 × 10^−02^	0.0071	1.4 × 10^−11^	0.0107	4.3 × 10^−04^	0.0007	7.5 × 10^−01^	0.0069	2.6 × 10^−01^
rs498475	28256240	G	0.366	0.989	0.0426	5.7 × 10^−09^	0.0564	7.3 × 10^−03^	0.0732	1.4 × 10^−12^	0.0694	2.4 × 10^−02^	0.0059	9.8 × 10^−09^	0.0107	3.6 × 10^−04^	0.0012	5.7 × 10^−01^	0.0082	1.7 × 10^−01^

Variant	BP^a^	EA^b^	EAF^c^	INFO^d^	BMI				Height				Weight			
					Discovery		Replication		Discovery		Replication		Discovery		Replication	
					Beta	*p*-value	Beta	*p*-value	Beta	*p*-value	Beta	*p*-value	Beta	*p*-value	Beta	*p*-value
rs10245867	28142186	T	0.329	0.995	−0.0079	7.6 × 10^−04^	−0.0057	4.1 × 10^−01^	0.0069	2.3 × 10^−06^	0.0146	1.6 × 10^−03^	−0.0029	1.5 × 10^−01^	0.0026	6.7 × 10^−01^
rs849138	28177338	G	0.493	0.998	−0.0108	1.1 × 10^−06^	−0.0054	4.0 × 10^−01^	0.0241	1.8 × 10^−69^	0.0361	1.7 × 10^−16^	0.0049	1.0 × 10^−02^	0.0134	2.0 × 10^−02^
7:28178625	28178625	TG	0.493	0.997	−0.0109	8.5 × 10^−07^	−0.0057	3.8 × 10^−01^	0.0240	4.7 × 10^−69^	0.0355	4.9 × 10^−16^	0.0048	1.2 × 10^−02^	0.0129	2.5 × 10^−02^
rs849333	28219812	A	0.342	0.996	−0.0082	4.1 × 10^−04^	−0.0051	4.5 × 10^−01^	0.0065	6.4 × 10^−06^	0.0163	3.8 × 10^−04^	−0.0031	1.2 × 10^−01^	0.0039	5.1 × 10^−01^
rs849335	28223990	T	0.341	N/A	−0.0078	7.5 × 10^−04^	−0.0062	3.6 × 10^−01^	0.0073	4.7 × 10^−07^	0.0152	9.1 × 10^−04^	−0.0024	2.4 × 10^−01^	0.0027	6.5 × 10^−01^
rs849336	28224053	A	0.341	1.000	−0.0078	8.2 × 10^−04^	−0.0062	3.6 × 10^−01^	0.0073	4.3 × 10^−07^	0.0151	9.8 × 10^−04^	−0.0023	2.5 × 10^−01^	0.0027	6.6 × 10^−01^
rs849327	28232457	A	0.341	0.997	−0.0078	8.7 × 10^−04^	−0.0058	3.9 × 10^−01^	0.0074	3.0 × 10^−07^	0.0149	1.2 × 10^−03^	−0.0022	2.7 × 10^−01^	0.0029	6.3 × 10^−01^
rs498475	28256240	G	0.366	0.989	−0.0055	1.7 × 10^−02^	−0.0035	6.0 × 10^−01^	0.0051	3.5 × 10^−04^	0.0127	4.9 × 10^−03^	−0.0019	3.5 × 10^−01^	0.0030	6.1 × 10^−01^

^a^ Base pair position in hg19 coordinates.

^b^ Effect allele.

c Effect allele frequency.

^d^ INFO, score for imputed genotypes provided by United Kingdom, Biobank. please note, no INFO, score is provided for directly genotyped variant(s).

Of note, WC not adjusted for BMI was not associated with any of the *JAZF1* SNPs, while BMI showed nominally significant associations with all eight SNPs, with the most significant association with the variant at position 7:28178625 which was also associated with WCadjBMI. Height also had a genome-wide significant association with the variant at position 7:28178625 and rs849138 and at least a nominal association with all other SNPs in this region whereas weight was not associated with any of the *JAZF1* SNPs except for a nominal association with the variant at position 7:28178625. These findings indicate that the association of the *JAZF1* SNPs with WCadjBMI was due to collider bias, when both the exposure and outcome are each causing another variable and that variable is adjusted for in the analysis. In this case, adjusting WC for BMI (collider) resulted in an association with the *JAZF1* SNPs (collider bias). This association was actually due to the *JAZF1* SNPs being associated with height, a component of BMI (Supplementary Figure S3).

To pursue these novel cross-phenotype associations, we analyzed the associations for these variants with all phenotypes in the replication dataset (Table 2). All variants identified as genome-wide significant in the discovery sample, were replicated with a *p*-value <0.05 in the replication dataset. For example, the variant at position 7: 28178625, had a replication *p*-value of 4.40 × 10^−2^ for asthma, 2.30 × 10^−3^ for T2D, and 1.70 × 10^−16^ for height.

Plots of the variants in the *JAZF1* region showed the variants most strongly associated with each phenotype were located near the transcriptional start site, first exon, and intron of this gene as well as in *JAZF1-AS1* (Figure 1). However, the association signal for each phenotype was stronger with variants in *JAZF1* rather than *JAZF1-AS1*, an observation confirmed by fine-mapping.

Fine-mapping of the *JAZF1* region identified one credible set for asthma, a second, non-overlapping credible set for T2D and four non-overlapping credible sets for height (Table 3). Since none of the variants in the credible sets across phenotypes were shared, our findings indicate different putative causal variants are associated with asthma, T2D, and height. All variants in the credible sets were in *JAZF1,* not *JAZF1-AS1*.

**TABLE 3:** Univariate fine-mapping results in the *JAZF1* region for asthma, T2D and height.

Variant	Position	A1^a^	AF^b^	INFO^c^	Association *p*-values			Asthma		T2D		Height	
					Asthma	T2D	Height	CS^d^	PIP^e^	CS^d^	PIP^e^	CS^d^	PIP^e^
7:27945054	27945054	CT	0.067	0.896	3.84E-01	6.24E-01	2.14E-16	None	0.0038	None	0.0034	HEIGHT_1	0.8047
rs773503722	27949022	CA	0.089	0.957	8.80E-01	3.00E-02	8.33E-13	None	0.0034	None	0.0047	HEIGHT_1	0.0248
rs12538288	27982312	C	0.059	0.984	9.47E-01	7.48E-02	1.12E-17	None	0.0034	None	0.0038	HEIGHT_1	0.0223
rs7783410	27982633	C	0.059	0.984	9.18E-01	6.25E-02	1.08E-17	None	0.0034	None	0.0039	HEIGHT_1	0.0233
rs4722746	27985368	C	0.059	0.983	9.08E-01	6.48E-02	2.00E-17	None	0.0034	None	0.0039	HEIGHT_1	0.0172
rs6970113	2,7991924	C	0.059	0.983	9.14E-01	7.68E-02	2.21E-17	None	0.0034	None	0.0038	HEIGHT_1	0.0167
rs1557779	27994758	T	0.072	0.984	9.68E-01	2.25E-02	1.18E-18	None	0.0034	None	0.0044	HEIGHT_1	0.0585
7:28154215	28154215	GT	0.792	0.979	3.03E-13	8.32E-05	4.57E-02	ASTHMA_1	0.0452	None	0.0034	None	0.0035
rs4722758	28156606	C	0.800	0.999	1.07E-13	6.68E-05	3.57E-02	ASTHMA_1	0.1135	None	0.0034	None	0.0033
rs6977955	28156887	C	0.800	0.999	1.61E-13	6.60E-05	3.79E-02	ASTHMA_1	0.0779	None	0.0034	None	0.0034
rs4719922	28158058	C	0.800	1.000	1.42E-13	6.44E-05	3.58E-02	ASTHMA_1	0.0877	None	0.0034	None	0.0033
7:28159795	28159795	GT	0.190	0.854	2.64E-01	1.13E-02	1.68E-57	None	0.0040	None	0.0033	HEIGHT_2	1.0000
rs9648346	28160113	C	0.800	1.000	1.50E-13	6.26E-05	3.58E-02	ASTHMA_1	0.0834	None	0.0034	None	0.0033
7:28168745	28168745	GTCTT	0.792	0.996	6.71E-14	2.68E-06	1.15E-02	ASTHMA_1	0.1410	None	0.0034	None	0.0028
rs2189965	28172014	C	0.800	1.000	3.01E-13	7.72E-05	3.06E-02	ASTHMA_1	0.0440	None	0.0034	None	0.0028
rs2189966	28172066	T	0.792	1.000	1.40E-13	4.59E-06	1.35E-02	ASTHMA_1	0.0711	None	0.0034	None	0.0034
rs4722760	28172183	A	0.800	0.999	3.07E-13	7.10E-05	2.93E-02	ASTHMA_1	0.0432	None	0.0034	None	0.0028
rs917115	28172586	T	0.792	0.999	1.36E-13	4.50E-06	1.44E-02	ASTHMA_1	0.0729	None	0.0034	None	0.0035
rs702814	28172732	C	0.494	0.997	8.97E-08	6.28E-18	1.25E-68	None	0.0052	T2D_1	0.0200	None	0.0017
rs917116	28172739	T	0.791	0.998	2.32E-13	4.30E-06	1.20E-02	ASTHMA_1	0.0454	None	0.0034	None	0.0033
rs67250450	28174986	T	0.800	0.999	3.06E-13	6.63E-05	3.05E-02	ASTHMA_1	0.0434	None	0.0034	None	0.0029
rs917117	28176305	G	0.801	0.998	1.94E-13	7.06E-05	3.67E-02	ASTHMA_1	0.0656	None	0.0034	None	0.0034
rs11495981	28177301	C	0.801	0.998	1.93E-13	7.09E-05	3.68E-02	ASTHMA_1	0.0660	None	0.0034	None	0.0034
rs864745	28180556	T	0.494	1.000	2.89E-07	3.93E-18	1.72E-70	None	0.0048	T2D_1	0.0286	None	0.0021
rs849142	28185891	T	0.494	1.000	4.75E-07	2.91E-18	7.77E-70	None	0.0046	T2D_1	0.0371	None	0.0018
rs11455969	28186775	A	0.494	0.999	3.79E-07	3.55E-18	1.12E-69	None	0.0047	T2D_1	0.0313	None	0.0018
rs10622246	28187111	A	0.498	0.994	5.96E-07	1.08E-18	3.08E-68	None	0.0046	T2D_1	0.0950	None	0.0020
rs849133	28192280	C	0.498	0.999	4.56E-07	7.26E-19	2.03E-71	None	0.0047	T2D_1	0.1330	None	0.0019
rs860262	28194397	C	0.498	0.999	5.75E-07	5.77E-19	1.46E-71	None	0.0046	T2D_1	0.1648	None	0.0020
rs849134	28196222	A	0.496	1.000	7.83E-07	1.84E-18	2.09E-70	None	0.0045	T2D_1	0.0575	None	0.0017
rs849135	28196413	G	0.499	1.000	6.09E-07	6.24E-19	1.61E-70	None	0.0045	T2D_1	0.1522	None	0.0016
rs1708302	28198677	C	0.499	0.999	6.07E-07	7.03E-19	6.77E-71	None	0.0045	T2D_1	0.1363	None	0.0017
rs1513272	28200097	C	0.499	0.999	5.92E-07	7.36E-19	6.69E-71	None	0.0046	T2D_1	0.1303	None	0.0017
rs537124	28203142	C	0.295	0.999	8.89E-01	1.49E-08	8.11E-112	None	0.0055	None	0.0033	HEIGHT_3	0.0309
rs552707	28205303	T	0.296	0.999	9.26E-01	1.39E-08	4.56E-112	None	0.0056	None	0.0033	HEIGHT_3	0.0493
rs543511926	28207300	G	0.999	0.828	6.85E-02	5.27E-01	4.23E-01	None	0.0056	None	0.0033	HEIGHT_3	0.0317
rs520161	28210660	T	0.299	1.000	8.67E-01	6.60E-09	1.91E-112	None	0.0052	None	0.0034	HEIGHT_3	0.5385
rs508347	28212824	T	0.299	0.999	8.14E-01	1.11E-08	3.53E-112	None	0.0050	None	0.0033	HEIGHT_3	0.3277
rs10226758	28214614	A	0.656	0.803	1.84E-05	5.52E-02	6.01E-24	None	0.0037	None	0.0037	HEIGHT_4	1.0000

^a^ Base pair position in hg19 coordinates.

^b^ Effect allele.

^c^ Effect allele frequency.

^d^ INFO, score for imputed genotypes provided by United Kingdom, Biobank. please note, no INFO, score is provided for directly genotyped variants.

^e^ 95% Credible Set.

^f^ Posterior Inclusion Probability.

To further explore the two genome-wide significant variants identified in the *JAZF1* region shared between asthma, T2D and height, we conducted mediation analyses in the discovery dataset with T2D as the outcome, mediated by either asthma or height (Table 4). The analysis of T2D mediated by asthma indicated that these two variants exhibited significant indirect effect through asthma, but that this mediation accounted for 5% or less of the total effect on T2D. A similar pattern was present for the mediation analysis through height; both variants had a significant indirect effect on T2D through height (Table 4), but the proportion mediated for rs849138 was negative due to the negative indirect effect of this variant on T2D through height. At the gene level, these mediation results are more consistent with biological rather than mediated pleiotropy given the small indirect effect.

**TABLE 4:** Mediation results for the two variants in *JAZF1* with cross-phenotype associations for asthma, T2D and height.

Variant	Total effect				Direct effect				Indirect effect				Proportion mediated			
	Beta	Lower CI^a^	Upper CI^a^	*p*-value	Beta	Lower CI^a^	Upper CI^a^	*p*-value	Beta	Lower CI^a^	Upper CI^a^	*p*-value	Estimate	Lower CI^a^	Upper CI^a^	*p*-value
	asthma-T2D															
rs849138	0.0048	0.0037	0.0059	<2e-16	0.0047	0.0036	0.0058	<2e-16	0.0001	0.0001	0.0002	<2e-16	0.0247	0.0156	0.0400	<2e-16
7:28178625	0.0046	0.0035	0.0057	<2e-16	0.0047	0.0037	0.0058	<2e-16	0.0001	0.0001	0.0002	<2e-16	0.0252	0.0133	0.0471	<2e-16
Height-T2D																
rs849138	0.0048	0.0037	0.0059	<2e-16	0.0051	0.0039	0.0061	<2e-16	−0.0003	−0.0003	−0.0002	<2e-16	−0.0523	−0.0759	−0.0400	<2e-16
7:28178625	0.0048	0.0037	0.0059	<2e-16	0.0045	0.0035	0.0056	<2e-16	0.0002	0.0002	0.0003	<2e-16	0.0488	0.0363	0.0700	<2e-16

^a^ 95% Confidance Interval.

## Discussion

Cross-phenotype associations can provide greater insight into correlated phenotypes, as well as elucidate underlying biological pathways. Anthropometric measures, asthma, and T2D are often correlated, and we used a homogenous subset of the United Kingdom Biobank to identify variants associated with all three phenotypes.

Across the phenotypes of interest, the only set of variants that are genome-wide significant outside of the HLA region for asthma, T2D, and at least one anthropometric measure are located in and around *JAZF1*. We focused our fine-mapping and mediation analyses on variants in this region.

Fine-mapping of the *JAZF1* region, suggests independent credible sets for each phenotype, with asthma and T2D each having one independent credible set and height having four credible sets which do not overlap with those for asthma or T2D (Table 3). This suggests independent causal variants for asthma, T2D, and height. A significant indirect effect was detected in the analysis of T2D mediated by asthma or height, this indirect effect represents less than 5% of the total effect. This small proportion of mediation through asthma or height likely not meaningful level (Table 4). The overlap of genetic signals are unlikely explained by mediation and these significant indirect effects are probably due to confounding from genetic correlation between traits and/or LD between the causal variants for each trait. The significance of the relatively small indirect effects of the variants on T2D either through asthma or height are driven primarily by the large sample size which make very small indirect effects detectable. Our goal in performing mediation analysis was not to estimate the proportion mediated, since it has been pointed out that there are substantial issues with this measure and its interpretation (Hayes, 2018), but to evaluate whether a substantial proportion of the total effect of the variant on T2D could be explained by its association with asthma or height. Here it was not, which supports a conclusion of gene-level biological pleiotropy.

*JAZF1* encodes a protein with three zinc fingers and acts as a transcriptional repressor. (Nakajima et al., 2004). It is member of a chaperone complex that orchestrates acetylation at regulatory regions controlling the expression of many genes involved in ribosome biogenesis. (Procida et al., 2021). Work on the *Jazf1* knockout mouse induced pluripotent stem cells suggests *JAZF1* is involved in differentiation of *β*-cells and glucose homeostasis. (Park et al., 2021). JAZF1 appears to limit inflammation in adipose tissue and mice overexpressing JAZF1 have lower body and fat weight. (Tighe et al., 1998). Finally, in mouse airway epithelial (Johnson et al., 2018) cultures, JAZF1 expression was shown to be necessary for multiciliated cell differentiation, which is important for removing contaminants from the airway. These functional studies suggest the plausibility of the role of *JAZF1* in asthma and T2D, but do not suggest a genetic link between these phenotypes.

Previous studies have found variants within *JAZF1* to be associated separately with T2D, obesity phenotypes, as well as, height. (Jang et al., 2014; Liao et al., 2019; Kobiita et al., 2020; Yengo et al., 2022). These findings include at least one study that reports a significant association with SNPs in *JAZF1* with WCadjBMI. (Christakoudi et al., 2021). Further, there is evidence *JAZF1* is associated with child-onset and possibly adult-onset asthma, (Ferreira et al., 2019; Laulajainen-Hongisto et al., 2020; Ma et al., 2020), demonstrating associations of variants in and around *JAZF1* with each of these phenotypes. However, this is the first time that asthma, T2D, and anthropometric measurements have been analyzed simultaneously in the same dataset. These findings also suggest that previous associations with SNPs in *JAZF1* with WCadjBMI are likely due to the same collider bias we observed, and the variants are associated with height, not adiposity. Additionally, this study is the first attempt at dissecting whether there are overlapping causal variants and/or biological pathways for these phenotypes and provides the strongest evidence for an association of variants in and around *JAZF1* with asthma.

The cohort data used here offers a unique opportunity to study cross-phenotype associations, as the phenotypes in question are available on almost all study subjects. This is particularly beneficial for dissecting overlapping signals, as mediation analyses largely rely on phenotypic and genetic data being available on the same subjects. Although methods are available that allow for mediation using summary statistics generated using different datasets, (Park et al., 2019), these approaches have not been sufficiently evaluated.

There is also the potential for misclassification of cases and controls for both asthma and T2D, but several measures were taken to limit any misclassification: 1) asthma and T2D were defined using data from both self-reported health questionnaires and ICD-10 codes; 2) inclusion criteria were based on the data collected during the first visit to ensure the same level of data collection across the study participants, given the different enrollment dates and varying number of follow-up visits across the UKB study; and 3) if an individual could be classified as a control for asthma or T2D at baseline but subsequently reported a diagnosis of asthma or T2D they were excluded.

When case-control studies are performed where individuals are ascertained based on a specific phenotype and then additional qualitative or quantitative phenotypes are analyzed that are correlated with the primary phenotype under study, biases can be introduced and type I and II errors increased. The United Kingdom Biobank was not ascertained on a particular phenotype, therefore we do not expect analyzing multiple phenotypes, even those that are correlated, will introduce biases. Meta-analysis methods have been proposed to detect pleiotropy that combine data across phenotypes to increase power and detect heterogeneity. When these methods are applied to summary statistics that contain overlapping subjects, this overlap needs to be taken into account, such as proposed in PLEIO. (Lee et al., 2021). We did not perform a meta-analysis because our univariate results showed genome-wide significant associations for all three phenotypes for variants in JAZF1 and performing meta-analysis would not increase our power to detect these cross phenotype associations.

While the United Kingdom Biobank offers substantial power to detect associations, one major limitation is that the majority of subjects are of White European ancestry with only a relatively small number of subjects of non-European ancestry, primarily of African and South Asian ancestry. Unfortunately, due to the limited sample sizes for individuals of African and South Asian ancestry, for many of the United Kingdom Biobank phenotypes, particularly for dichotomous phenotypes, the power to detect association is low. Future efforts should be made to study the reported associations in individuals of sufficiently powered studies of non-European ancestry to determine if the same patterns are observed.

Conclusion: Asthma and T2D are comorbidities which are also correlated with anthropometric measurements. *JAZF1* was previously shown to be associated with these phenotypes. Here we show that JAZF1 is likely associated with height and not adiposity. We also show that for asthma, T2D, and height, the association with JAFZ1 is due to biological pleiotropy on the gene-level but likely due to different causal variants underlying these phenotypes.

Regional plots were created using LocusZoom (Boughton et al., 2021) from the discovery association results for asthma (A), type 2 diabetes (B), and height. The most significantly associated variant for each of the three phenotypes, was selected as the reference variant and is indicated with purple diamond in each plot. Linkage disequilibrium (as measured by *r* ^2^) was estimated from the data. It is evident from each plot that the strongest association signal for each phenotype is within *JAZF1* rather than *JAZF1-AS1*.

Pairwise linkage disequilibrium (as measured by *r* ^2^) is displayed for the eight genome-wide significant variants (see Table 2). Where an *r* ^2^ value is not indicated, *r* ^2^ = 1.0. The plot was created in HaploView (Barrett et al., 2005) using the subset of unrelated subjects.

## Data Availability

The datasets presented in this article are not readily available because all researchers who wish to access the research resource must register with UK Biobank by completing the registration form. Requests to access the datasets should be directed to https://www.ukbiobank.ac.uk/.

## Abbreviations

BMI, body mass index; GLMM, generalized linear mixed models; HLA, human leukocyte antigen; ICD, international classification of disease; JAZF1, juxtaposed with another zinc finger protein 1; JAZF1-AS1, JAZF1 antisense RNA 1; LD, linkage disequilibrium; LMM, linear mixed models; MAF, minor allele frequency; PC, principal component; T2D, Type 2 Diabetes; WC, waist circumference and WC adjusted for BMI (WCadjBMI).
