# Supplementary material for: Variants in JAZF1 are associated with asthma, type 2 diabetes, and height in the United Kingdom biobank population
Source: Front Genet. 2023 Jun 12;14:1129389. doi: 10.3389/fgene.2023.1129389 (PMC10291233; doi:10.3389/fgene.2023.1129389)
Supplement: Supplementary file 1 [file Table1.DOCX]

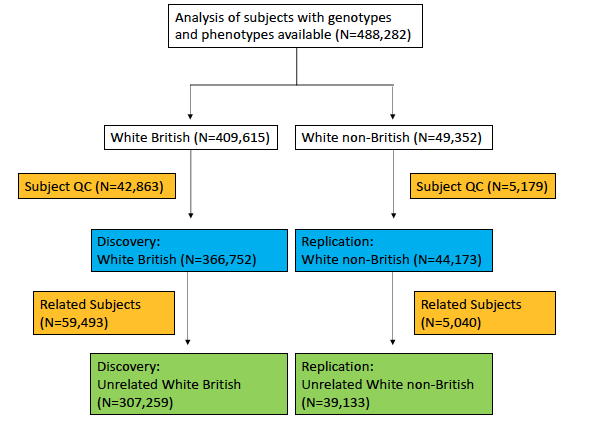


Figure S1: Summary flowchart describing the creation of the full Discovery and Replication datasets (blue), as well as the unrelated Discovery and Replication datasets (green)_used for the mediation analyses, based on the QC in Tables S1 and S2.

A


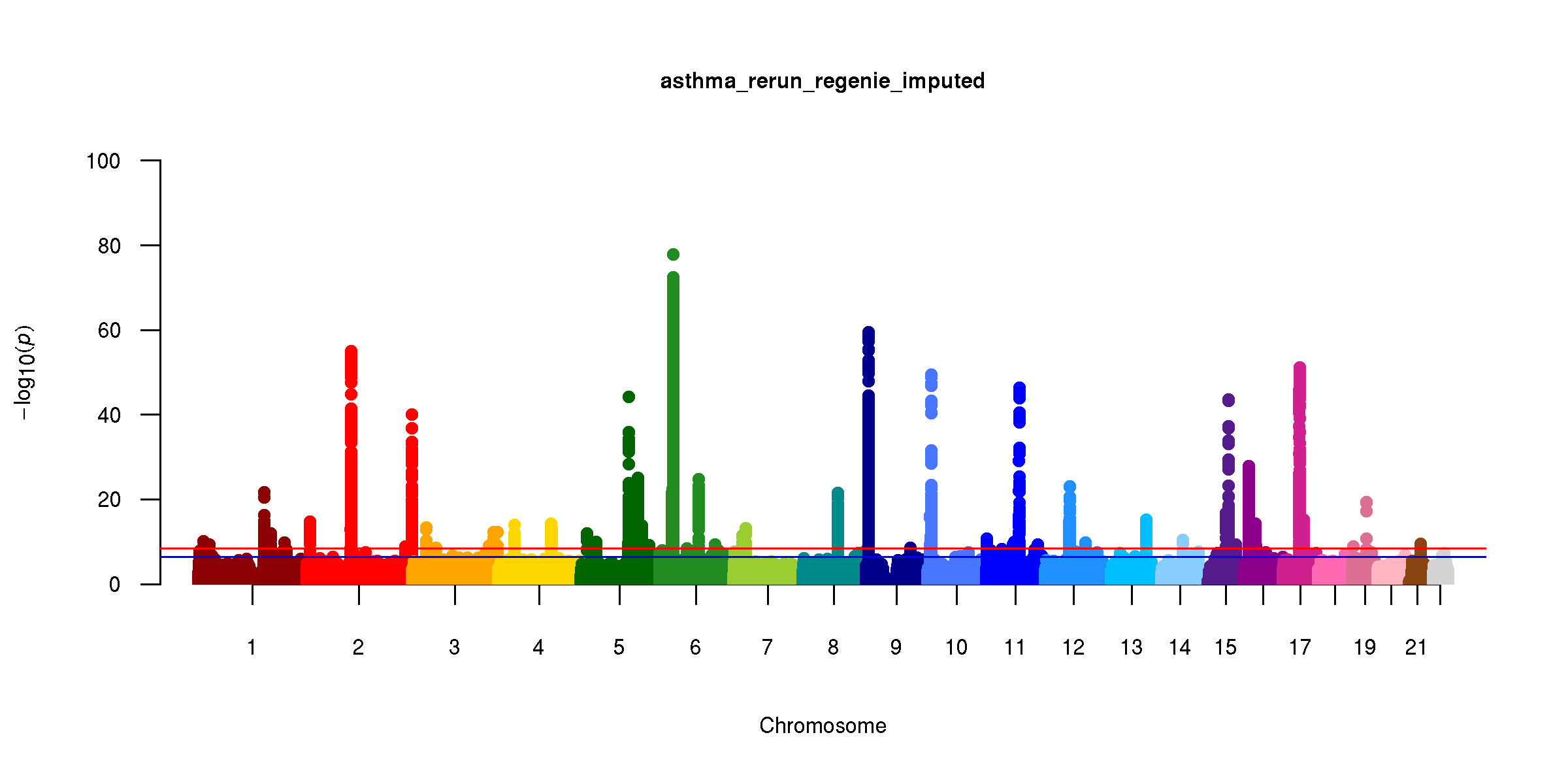


A


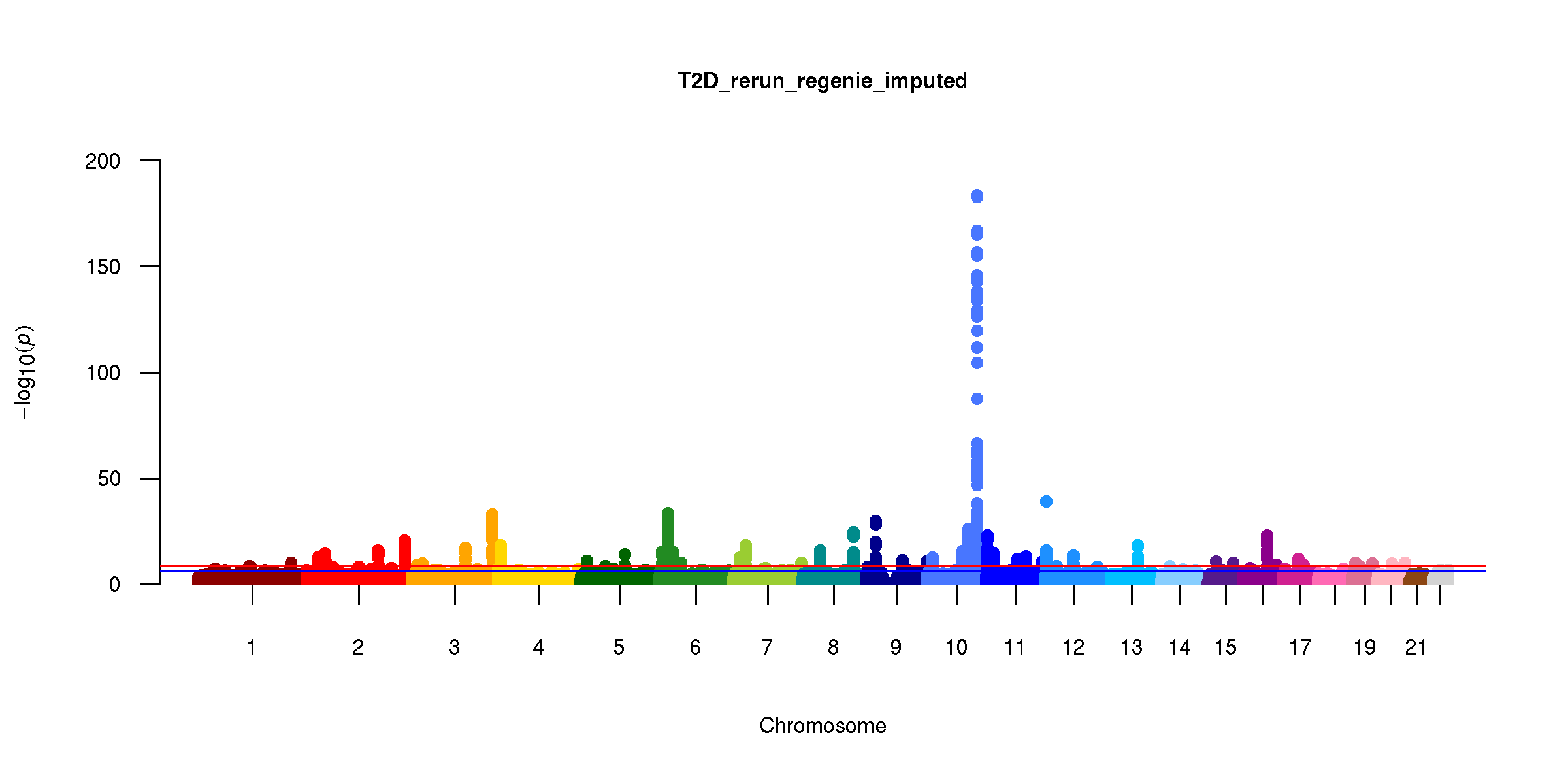


A


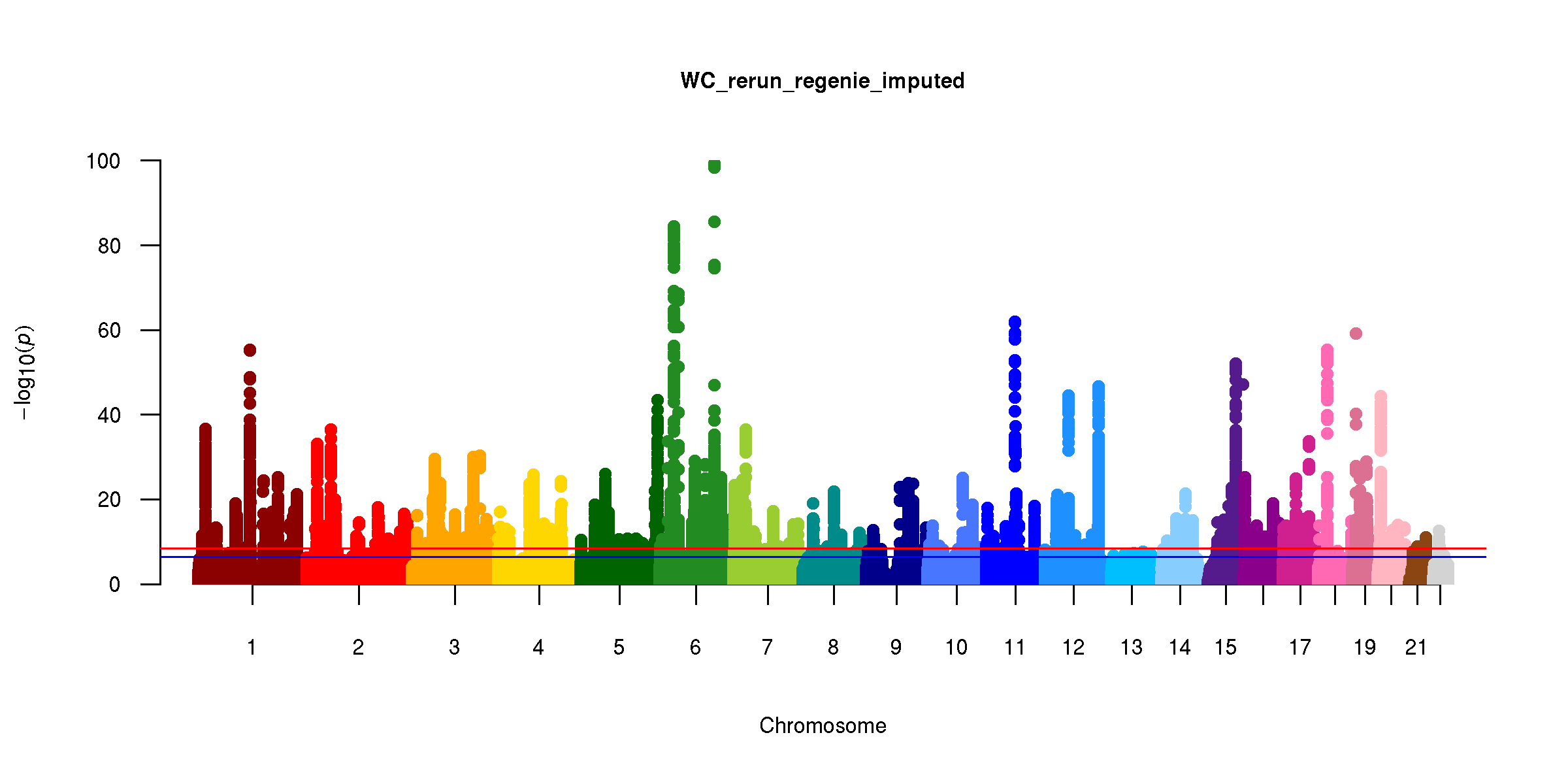


A

D.


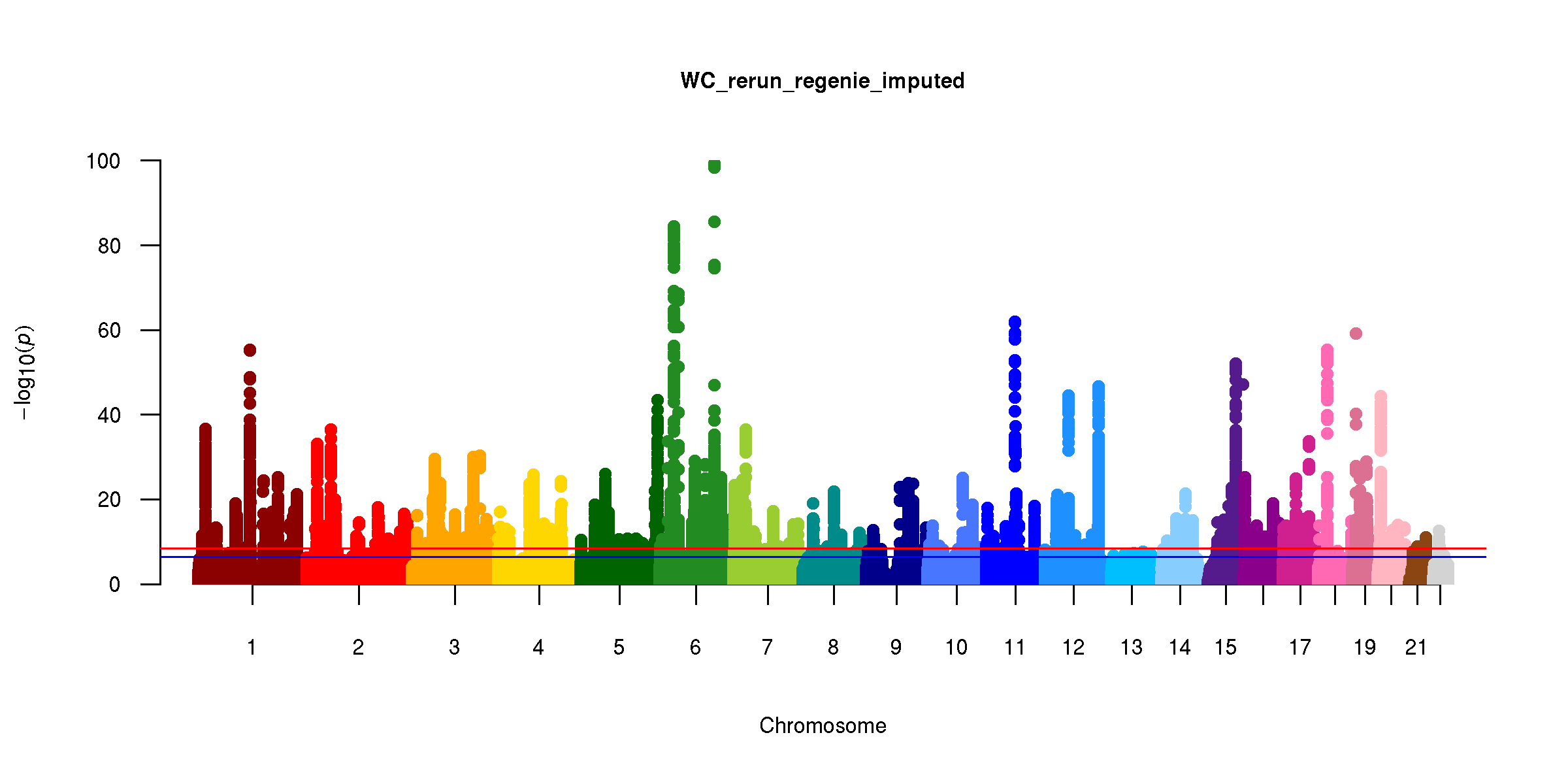


A

E.


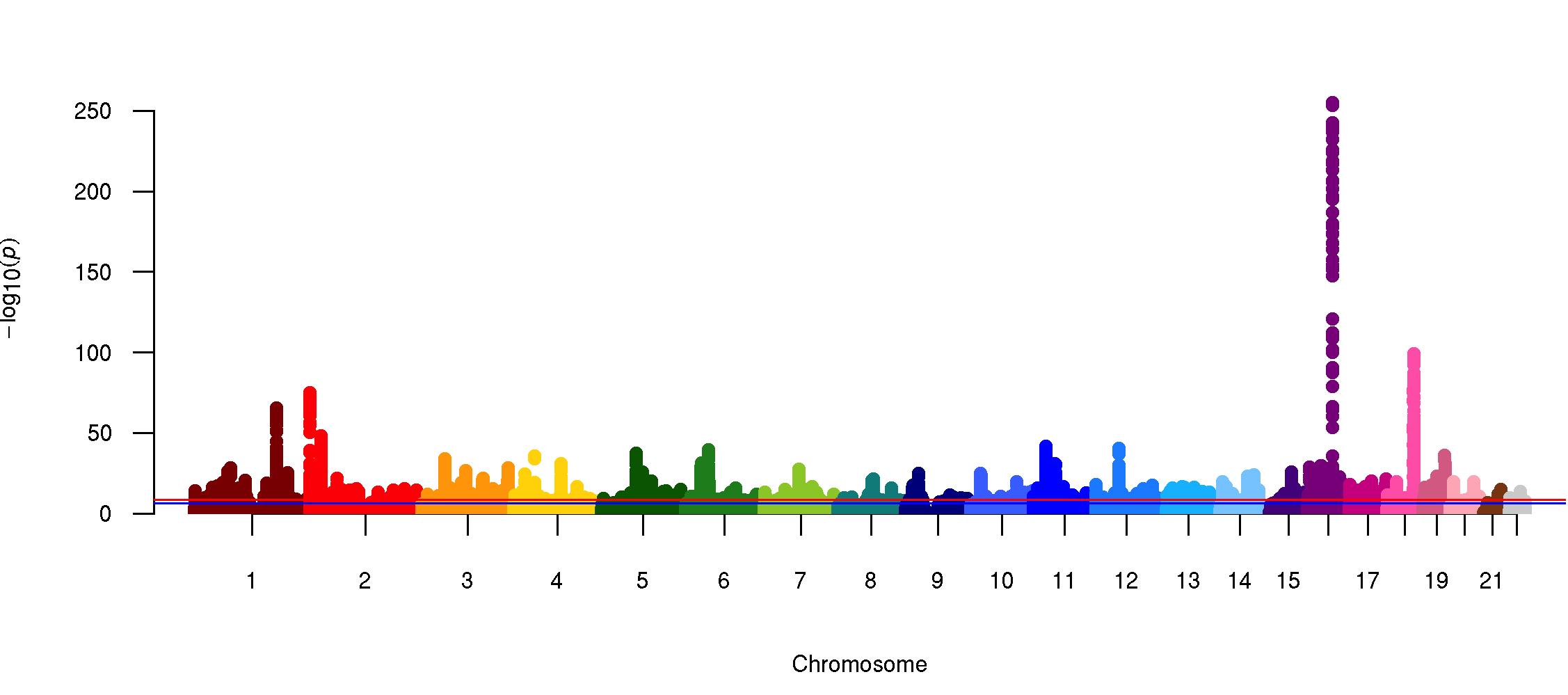


F.


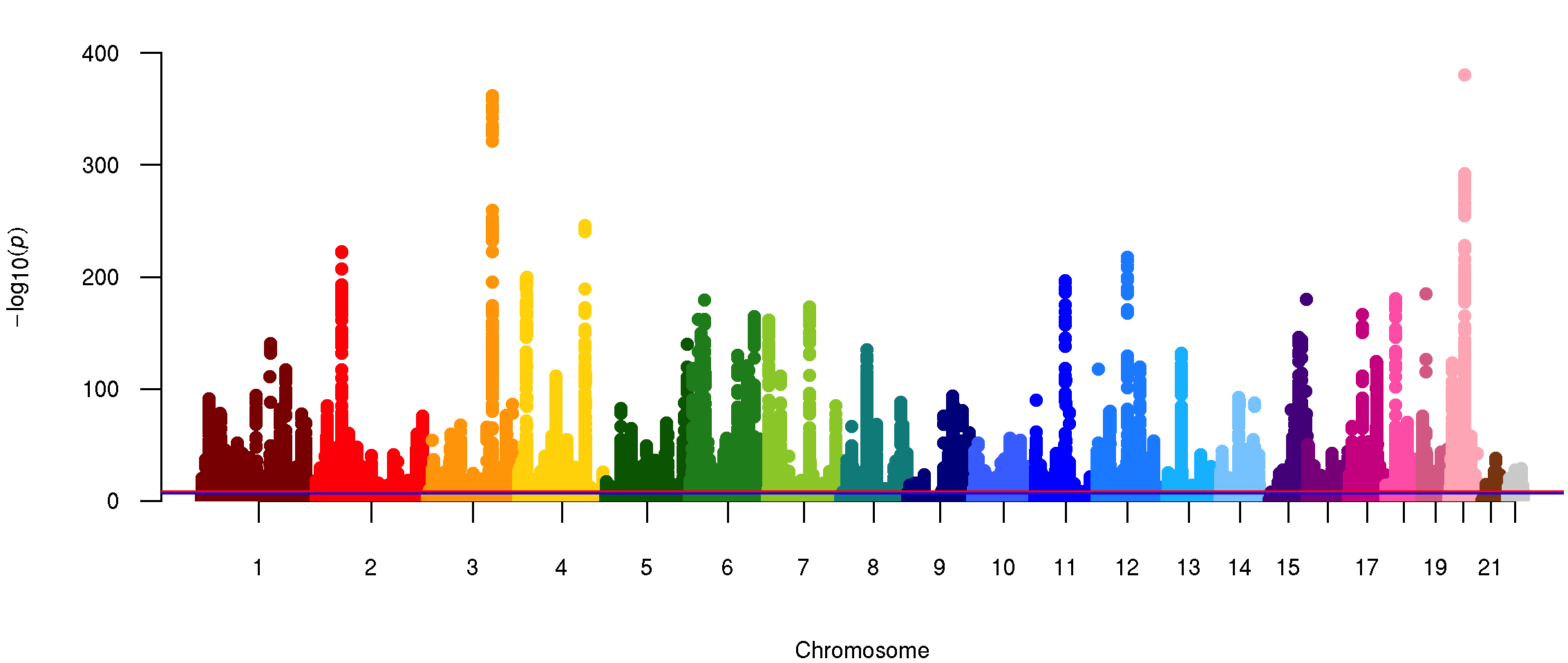


G.


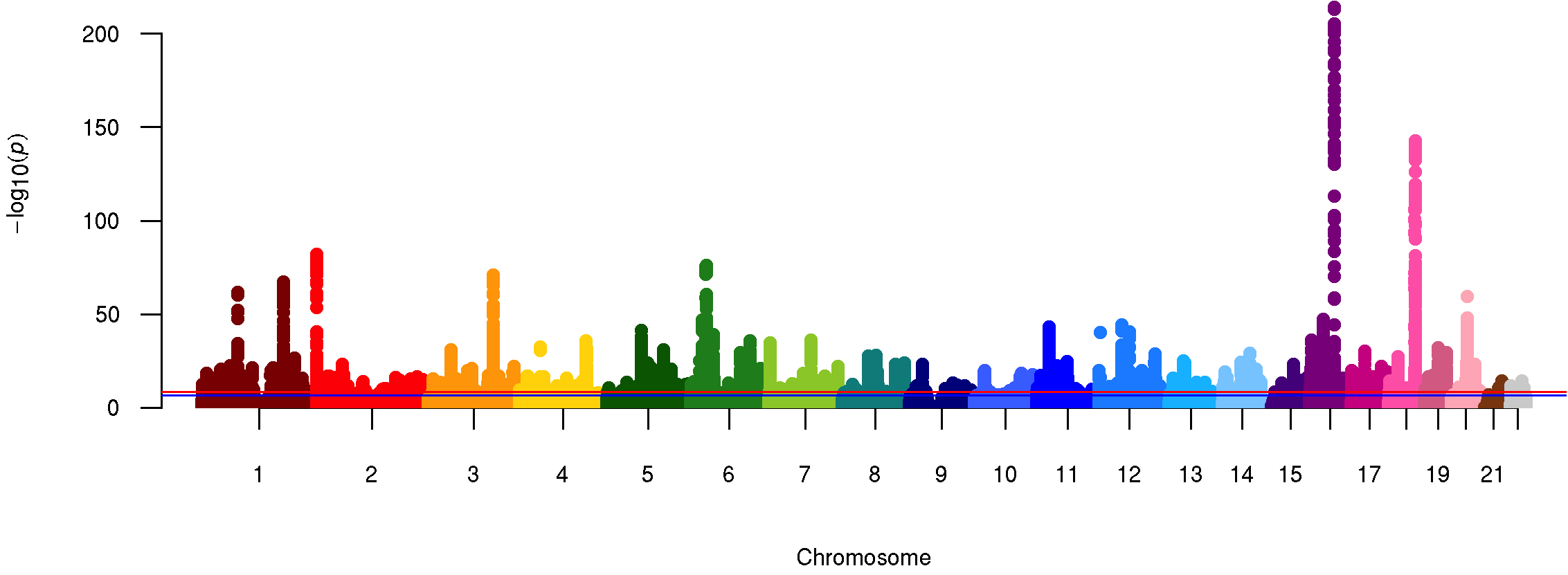


Figure S2: Manhattan plots for asthma (A), type 2 diabetes (B), waist circumference adjusted for BMI (C), waist circumference not adjusted for BMI (D), BMI (E), height (F) and weight (G) for the discovery sample. Genome-wide significant threshold (p=5.0x10^-8^) is indicated by the red line and genome-wide suggestive threshold (5.0x10^-6^) is indicated by the blue line.


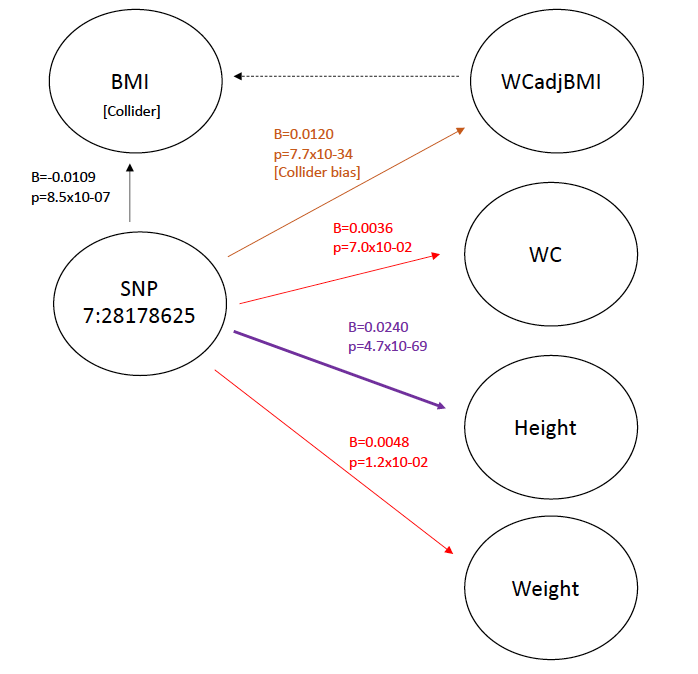


Figure S3: Diagram of the associations between SNP 7:28178625 and the major phenotypes examined. The association with WCadjBMI is due to the adjustment for BMI which is a collider due to the strong association with height, one aspect of BMI, shown in purple. Both weight and WC (unadjusted) did not show association with this SNP.

Table S1: Discovery quality control

|  | # variants | # variants removed in this step | # subject | # subjects removed in this step |
| --- | --- | --- | --- | --- |
| Step 1: Initial variant QC |  |  |  |  |
| Genotyped variants | 805,426 |  |  |  |
| Autosomal variants | 784,256 | 21,170 |  |  |
| Covered by both arrays | 733,322 | 50,934 |  |  |
| Batch level qc | 687,004 | 46,318 |  |  |
| SNPs only (indels removed) | 674,489 | 12,515 |  |  |
|  |  |  |  |  |
| Step 2: Subject QC1 |  |  |  |  |
| Genotypes available |  |  | 488,377 |  |
| Phenotypes available |  |  | 488,282 | 95 |
| Genetic and reported sex match |  |  | 487,910 | 372 |
| Sex chromosomes non-XX XY |  |  | 487,440 | 470 |
| Outliers in heterozygosity/missing rate |  |  | 486,477 | 963 |
| "Caucasian" (f.22006) |  |  | 408,186 | 78,291 |
| Individual call rate > 99% |  |  | 366,752 | 41,434 |
| Unrelated |  |  | 307,259 | 59,493 |
|  |  |  |  |  |
| Step 3: Final variant QC2 |  |  |  |  |
| call rate > 99% | 639,862 | 34,627 |  |  |
| HWE p<5x10-8 | 610,019 | 29.843 |  |  |
| MAF > 1% | 529,024 | 80,995 |  |  |
|  |  |  |  |  |
| Total | **529,024** |  | **366,752** |  |
|  |  |  |  |  |
| 1Subject QC was performed using the 674,489 variants | | |  |  |
| 2Unrelated subjects were used for final variant QC and then the set of variants were selected for the full set of subjects | | | | |

Table S2: Replication quality control

|  | # variants | # variants removed in this step | # subjects | # subjects removed in this step |
| --- | --- | --- | --- | --- |
| Step 1: Initial variant QC |  |  |  |  |
| Genotyped variants | 805,426 |  |  |  |
| Autosomal variants | 784,256 | 21,170 |  |  |
| Covered by both arrays | 733,322 | 50,934 |  |  |
| Batch level qc | 687,004 | 46,318 |  |  |
| SNPs only (indels removed) | 674,489 | 12,515 |  |  |
|  |  |  |  |  |
| Step 2: Subject QC^1^ |  |  |  |  |
| Mahalanobis defined non-British white |  |  | 49,352 |  |
| Genetic and reported sex match |  |  | 49,307 | 45 |
| Sex chromosomes non-XX XY |  |  | 49,265 | 42 |
| Outliers in heterozygosity/missing rate |  |  | 49,133 | 132 |
| Individual call rate > 99% |  |  | 44,173 | 4,960 |
| Unrelated^2^ |  |  | 39,133 | 5,040 |
|  |  |  |  |  |
| Step 3: Final variant QC2 |  |  |  |  |
| call rate > 99% | 639,847 | 34,642 |  |  |
| HWE p<5x10-8 | 631,089 | 8758 |  |  |
| MAF > 1% | 550,028 | 81,061 |  |  |
|  |  |  |  |  |
| Total | **550,028** |  | **44,173** |  |
|  |  |  |  |  |
| ^1^Subject QC was performed using the 674,489 variants | | | | |
| ^2^Unrelated subjects were used for final variant QC and then the set of variants were selected for the full set of subjects | | | | |
